# SOX4 Transcriptionally Regulates Multiple SEMA3/Plexin Family Members and Promotes Tumor Growth in Pancreatic Cancer

**DOI:** 10.1371/journal.pone.0048637

**Published:** 2012-12-12

**Authors:** Hsin-Yi Huang, Yu-Yao Cheng, Wei-Chih Liao, Yu-Wen Tien, Chih-Hsin James Yang, Su-Ming Hsu, Pei-Hsin Huang

**Affiliations:** 1 Graduate Institute of Pathology, College of Medicine, National Taiwan University, Taipei, Taiwan, Republic of China; 2 Department of Pathology, National Taiwan University Hospital, Taipei, Taiwan, Republic of China; 3 Department of Internal Medicine, National Taiwan University Hospital, Taipei, Taiwan, Republic of China; 4 Department of Surgery, National Taiwan University Hospital, Taipei, Taiwan, Republic of China; 5 Department of Oncology, National Taiwan University Hospital, Taipei, Taiwan, Republic of China; Technische Universität München, Germany

## Abstract

Semaphorin signaling through Plexin frequently participates in tumorigenesis and malignant progression in various types of cancer. In particular, the role of semaphorin signaling in pancreatic ductal adenocarcinoma (PDAC) remains unexplored, despite a high likelihood of metastasis and mortality. Unlike other epithelial malignancies that often express a small number of specific genes in the Semaphorin/Plexin family, five or more are often expressed in human PDAC. Such concomitant expression of these SEMA3/Plexin family members is not a result of gene amplification, but (at least partially) from increased gene transcription activated by SOX4 de novo expressed in PDAC. Via chromatin-immunoprecipitation, luciferase promoter activity assay and electrophoresis mobility shift assay, SOX4 is demonstrated to bind to the consensus site at the promoter of each *SEMA3* and *Plexin* gene to enhance transcription activity. Conversely, RNAi-knockdown of SOX4 in PDAC cell lines results in decreased expression of SEMA3/Plexin family members and is associated with restricted tumor growth both *in vitro* and in SCID mice. We further demonstrate that SOX4 levels parallel with the summed expression of SEMA3/Plexin family members (*P* = 0.033, *NPar* Kruskal-Wallis *one*-*way* analysis), which also correlates with poor survival in human PDAC (*P* = 0.0409, *Kaplan-Meier* analysis). Intriguingly, miR-129-2 and miR-335, both of which target SOX4 for degradation, are co-repressed in human PDAC cases associated with up-regulated SOX4 in a statistically significant way. In conclusion, we disclose a miR-129-2(miR-335)/SOX4/Semaphorin-Plexin regulatory axis in the tumorigenesis of pancreatic cancer.

## Introduction

Pancreatic ductal adenocarcinoma (PDAC) is the fourth leading cause of cancer death in the United States, and one of the ten most common cancers in Taiwan. Diagnosis of PDAC often occurs only after widespread metastasis. As a result, the poor prognosis of PDAC can be attributed to its aggressive biological behavior and its resistance to chemotherapy or radiotherapy, which necessitates a detailed mechanistic study of PDAC for therapeutic design [1].

Gene expression and genome-wide mutational studies indicate that human pancreatic cancer results from alteration of multiple genes that function through a core set of at least 12 cellular signaling pathways [2]. Through an analysis of more than 20000 transcripts, an average of 63 genetic alterations in pancreatic cancer has been demonstrated. The mechanisms for aberrant expression of each specific gene in pancreatic cancer are diverse, including point mutation, gene deletion, and amplification [2].

The aberrant expression of class 3 Semaphorin as well as the cognate receptors Plexin (PLXN) and Neuropilin (NRP) in different types of human cancer indicates that SEMA3-gated Plexin signaling regulates cancer cell behaviors [3], [4]. For example, over-expression of SEMA3C and SEMA3E mediates tumor progression and metastasis in cancers, including human lung adenocarcinoma, prostate cancer, endometrioid cancer, and mammary adenocarcinoma. By contrast, the *SEMA3F* and *SEMA3B* genes are frequently deleted in epithelial malignancy, resulting in enhanced angiogenesis [3]. For PDAC, over-expression of NRP1 and SEMA3A has been reported to correlate with a poor prognosis [5]. However, the molecular processes underlying such expression during PDAC tumorigenesis have not been elucidated, and whether other members of class 3 Semaphorin or Plexin participate in pancreatic cancer formation or progression is not yet known. Here, we examined the expression of SEMA3 and Plexin/Neuropilin in normal human pancreas, PDAC surgical specimens, and PDAC cell lines. Unlike other epithelial malignancies that often specifically over-express a small number of genes in the Semaphorin family, most PDAC cases over-express more than five genes related to SEMA3 and Plexin. The mechanism underlying the co-expression of SEMA3/Plexin family members in PDAC was thus investigated.

## Materials and Methods

### Ethics Statement

We confirmed that work on human tissue blocks and fresh paired tumor/non-tumor samples in this study was approved by the Tissue and Ethics Committee, with written informed consents obtained following the guidelines set forth by Tissue and Ethics Committee at NTUH. The *in vivo* tumorigenesis assay in SCID mice was performed under an approval issued by the Institutional Animal Care and Use Committee (IACUC) of National Taiwan University College of Medicine and College of Public Health.

### Case selection

Sixty-two cases of pancreatic ductal adenocarcinoma were identified via a search of the pathological records registered in National Taiwan University Hospital (NTUH), Taiwan from January 1996 to December 2006. For quantitative real-time PCR, twenty-three cases of paired snap-frozen pancreatic non-tumor and tumor tissues were obtained. Selected demographic information was retrieved from the hospital cancer registry as detailed in Supplementary Table S1.

### Cell culture, RNA interference, and selection of stable-transfected cell clones

PANC-1, MiaPaCa2, and Capan-1 cells were purchased from ATCC. All cells were grown in DMEM medium supplemented with 10% fetal bovine serum and 1% penicillin/streptomycin at 37°C with 5% CO_2_.

For RNA interference, the targeting oligonucleotides specific for *SOX4* gene (NM_003107.2: nt.1999–2019 and nt.1362–1382) or scrambled sequences were cloned in the pSuperGFP/neo vector. Lentiviral shRNAs targeting SOX4 via the constructed oligonucleotides directed against nt.4433–4453 and nt.1101–1121 (NM_003107.2), respectively, were purchased from RNAi core laboratory in Taipei Sinica Academica. *PLXNA2* siRNA plasmids were commercially purchased (Santa Cruz). The stably plasmid-transfected clones were selected by G418 (Sigma). RNAi-knockdown of each molecule was confirmed by RT-PCR and Western blot.

### Antibodies, immunohistochemistry, and Western blot

The antibodies used included anti-human NRP1 (R&D Systems), SEMA3A, -3B, -3C, -3E, PLXNA1, -A2, -A3, -D1, NRP2, SOX4 (Santa Cruz), SEMA3F (Chemicon), Histone 3 (Millipore), Ki-67 (DAKO), ERK1/2, phospho-ERK1/2 (Signal Transduction), PCNA, and tubulin (Calbiochem). Immunohistochemistry and Western blot were performed as previously described [6].

### Soft agar clonogenic assay, flow cytometry, BrdU labeling, and in vivo tumorigenesis assay

These assays were performed as described previously [7].

### Quantification of mRNA or micro-RNA and chromatin immunoprecipitation assay (ChIP)

ChIP assays were performed using the EZ Magna Kit (Millipore) as indicated by the manufacturer. Real-time PCR analysis was used to quantify mRNA or micro-RNA as previously described [6]. Primers used were detailed in Supplementary Materials and methods.

### Array comparative genomic hybridization

DNA for array comparative genomic hybridization was extracted from PANC-1, MiaPaCa2 cells or normal human female genomic DNA. Cell line DNA and control DNA were labeled with Cy5 or Cy3, respectively, from duplicate experiments and hybridized to array slides containing 2621 BAC clones at an average 1-Mbp resolution (SpectralChip 2600 array, PerkinElmer). Slides were scanned for Cy3 and Cy5 fluorescence using the GenePix® 4000B Microarray Scanner (Molecular Devices). Images were analyzed using SpectralWare 2.2 (Spectral Genomics).

### Detection of K-Ras and SOX4 gene mutation

Tumor sections in the retrieved formalin-fixed paraffin-embedded human tissues were dissected and de-paraffinized, and genomic DNA was extracted. All primers used have been noted in Supplementary Materials and methods.

### Luciferase reporter assay

The predicted promoter sequences were cloned into luciferase reporter vector pGL3Basic (Promega) and verified by direct sequencing. HeLa cells in 24-well plates were co-transfected with the indicated construct plus a SOX4-expressing construct and pRL-TK plasmid DNA using Lipofectamine 2000 (Invitrogen). After transfection for 48 hours, cells were harvested for a luciferase activity assay (Promega).

### Electrophoretic mobility shift assay (EMSA)

Nuclear extract was prepared from HeLa cells and the total protein concentration determined by Bradford reagent (BioRad). For each EMSA experiment, 2000 cpm of ^32^P-labeled double stranded DNA fragments was used for a binding reaction containing nuclear extract and poly-dI-dC (Sigma) at 30°C for 30 min in a solution containing 5 mM MgCl_2_, 20 mM HEPES-KOH pH 7.9, 60 mM KCl, 1 mM DTT, 0.5 mg/ml bovine serum albumin, 12% glycerol and 1 mM PMSF. In the competition assay, five- or twenty-fold volume of cold probe relative to hot probe was used. After stopping the binding reaction, samples were subjected to gel electrophoresis, vacuum-dried, and exposed to X-ray film for radioactivity detection.

### Statistical analysis

A paired *t*-test test was used to assess the correlation between the results of immunohistochemical staining in tumor and non-tumor tissues. The impact of the immunohistochemistry results on the overall survival of patients with PDAC was assessed by Kaplan-Meier survival analysis, and the differences between sub-groups were analyzed by log-rank test. A one-way NPar test was used to assess the association between SOX4 (or E2F1) expression and the immunohistochemistry intensity of SEMA3, NRP, or Plexins. Pearson correlation was used to assess the relationship between normalized SOX4 expression and microRNA level (presented as fold changes in which the ratio of the value derived from tumor versus that derived from non-tumor part in paired fresh tissues was used). A log transformation (base 10) of each ratio value was performed to achieve the normal distribution required for the linear regression test. Statistics were obtained using SPSS 12.0 software (SPSS), and calculations on patient survival were performed using GraphPad Prism 4.0 software (GraphPad software).

## Results

### Concomitant expression of multiple members of class 3 Semaphorins and the receptor complex- Neuropilin/Plexin family in PDAC

**Figure 1:**
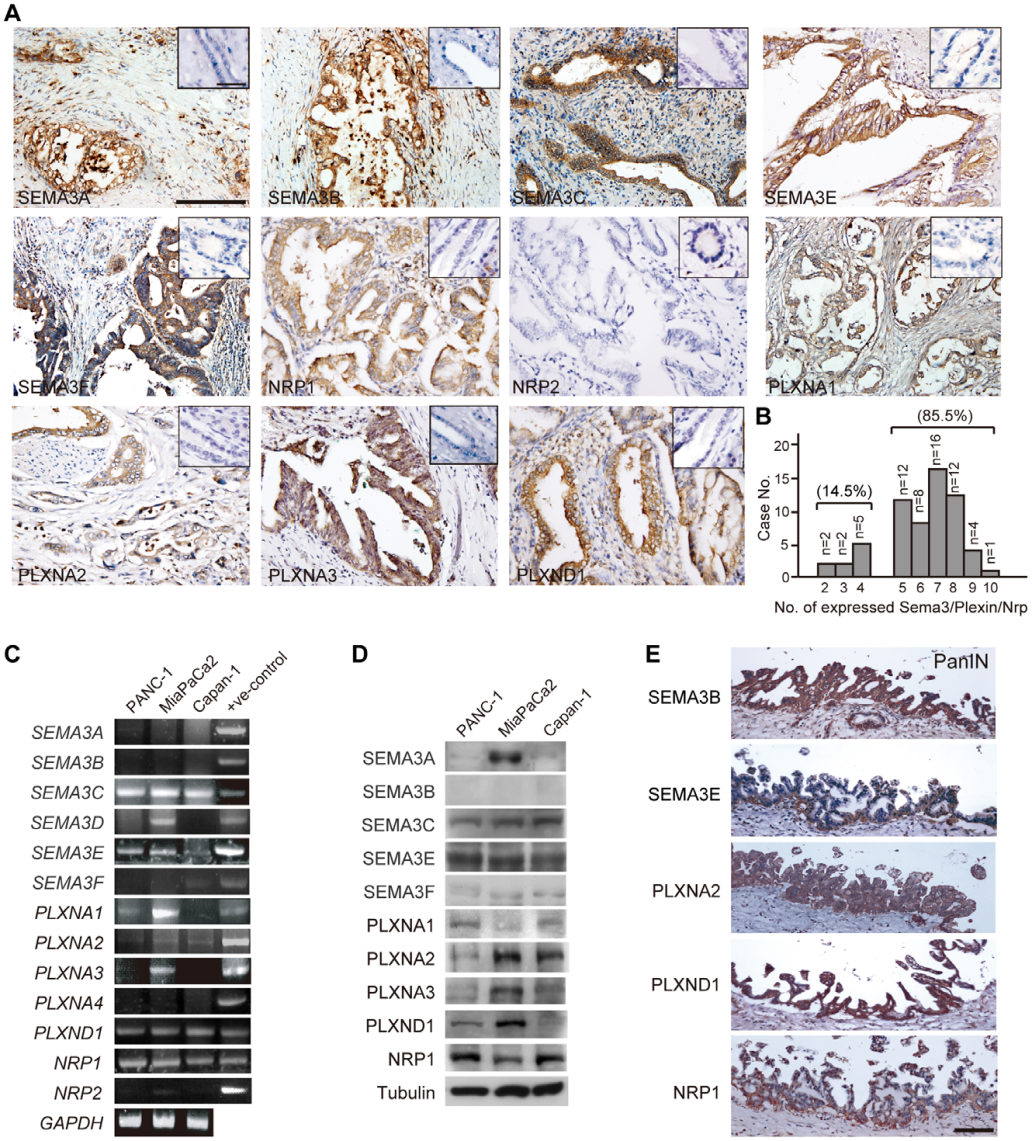
Multiple members of SEMA3/Plexin ligand-receptor complexes expressed in human PDAC tissues and cell lines. (A) immunohistochemistry of human PDAC cases showing expression of NRP1 and most members of SEMA3/Plexin family. Normal pancreatic tissue did not express any SEMA3/Plexin/NRP (diagonal, scale bar = 50 µm). Scale bar = 100 µm. (B) Bar graph to show distribution of numbers of SEMA3/Plexin/NRP expressed in each PDAC case. Fifty-three out of 62 cases (85.5%) expressed more than 5 members. (C) semi-quantitative RT-PCR analysis of SEMA3-, Plexin- and NRP- transcripts in human PDAC cell lines. GAPDH served as an internal loading control. (D) immunoblot of the whole-cell lysate from human PDAC cell lines to detect endogenous SEMA3, Plexin, and NRP1. (E) representative SEMA3-, Plexin- and NRP1-immunoreactivity detected in PanIN lesions adjacent to invasive carcinoma. Scale bar = 100 µm.

To investigate whether SEMA3 or the related receptors (Plexin/Neuropilin) are involved in the carcinogenesis of pancreatic cancers, immunohistochemistry was performed in 62 PDAC cases paired with normal pancreatic tissue. As shown in Fig. 1A, normal pancreatic ductal and acinar epithelial cells do not express SEMA3 or Plexin. By contrast, pancreatic adenocarcinoma cells showed immunoreactivity toward most members of SEMA3 and Plexin family, and NRP1 (Fig. 1A). More than 90% of PDAC cases revealed strong cytoplasmic or membranous immunostaining of SEMA3E, and a variable ratio (38%∼68%) of cases were immunoreactive toward SEMA3A, SEMA3B, SEMA3C and SEMA3F (Table 1). As for SEMA3 receptors, PLXND1 and NRP1 were detectable in most PDACs, accounting for 90% of the cases. By contrast, NRP2 was much less frequently expressed in PDACs (accounting for 14%), and there were variable expression ratios of PLXNA1, PLXNA2 and PLXNA3 (31.67%, 68.85% and 66.67%, respectively; Table 1). When the combined expression of all SEMA3, PLXN, and NRP1 members in each PDAC case was evaluated, it became evident that most PDAC cases (accounting for 85.5% in our samples) expressed 5–10 members of SEMA3/Plexin family (Fig. 1B). In line with the co-expression of SEMA3/Plexin in PDAC surgical specimens, human PDAC-derived cell lines (PANC1, Capan1 and MiaPaCa2 cells) also expressed multiple members of SEMA3, Plexin and NRP1 at the transcript and protein levels (Fig. 1C and 1D).

**Table 1:** Expression of SEMA3, Plexin and NRP in patients with PDAC.

Molecule	Case	Immuno-positivity (number, %)	
	No.	Tumor	Ductal epithelia
SEMA3A	59	32 (54.24%),**	0 (0%)
SEMA3B	60	41 (68.33%),**	0 (0%)
SEMA3C	62	24 (38.71%),**	0 (0%)
SEMA3E	62	58 (93.55%),**	0 (0%)
SEMA3F	59	28 (47.46%),**	0 (0%)
PLXNA1	60	19 (31.67%),**	0 (0%)
PLXNA2	61	42 (68.85%),**	0 (0%)
PLXNA3	57	38 (66.67%),**	0 (0%)
PLXND1	62	57 (91.94%),**	0 (0%)
NRP1	62	57 (91.94%),**	0 (0%)
NRP2	62	9 (14.52%),**	0 (0%)

Paired *t*-test,

** : *P*<0.0001.

When Kaplan-Meier survival analysis was performed in correlation with the expression of SEMA3, Plexin or NRP members, no significant association was found except that SEMA3F-positive cases displayed longer survival and less frequent lymph node metastasis (Supplementary Fig. S1 and unpublished data). In addition, no correlation was observed between nodal metastasis and expression level of SEMA3 or Plexin (Supplementary Table S2). Moreover, RNAi-mediated knockdown of a single SEMA3 or Plexin resulted in an insignificant effect on both cell survival and proliferation (Supplementary Fig. S2B, S2C, S2D). Consequently, no single member of SEMA3/Plexin plays a crucial role in tumor formation or progression of PDAC.

Given that pancreatic cancer expresses variable amounts of SEMA3 and Plexin in strong contrast to non-expression in normal pancreatic ductal tissue, we speculated that concurrent expression of SEMA3/Plexin might be involved in PDAC tumorigenesis. Therefore, we next examined the expression of SEMA3 and Plexin in pancreatic precursor cancerous lesions, specifically, pancreatic intraepithelial neoplasia (PanIN). As shown in Fig. 1E, multiple members of SEMA3 and Plexin plus NRP1 were already present in PanIN lesions adjacent to invasive ductal neoplastic tumor nests, suggesting that concomitant expression of SEMA3/PLXN/NRP1 occurs in the early steps of PDAC tumorigenesis.

### Expression of SOX4 in PDAC correlates with the summed expression of genes from the NRP1, SEMA3 and Plexin families

**Figure 2:**
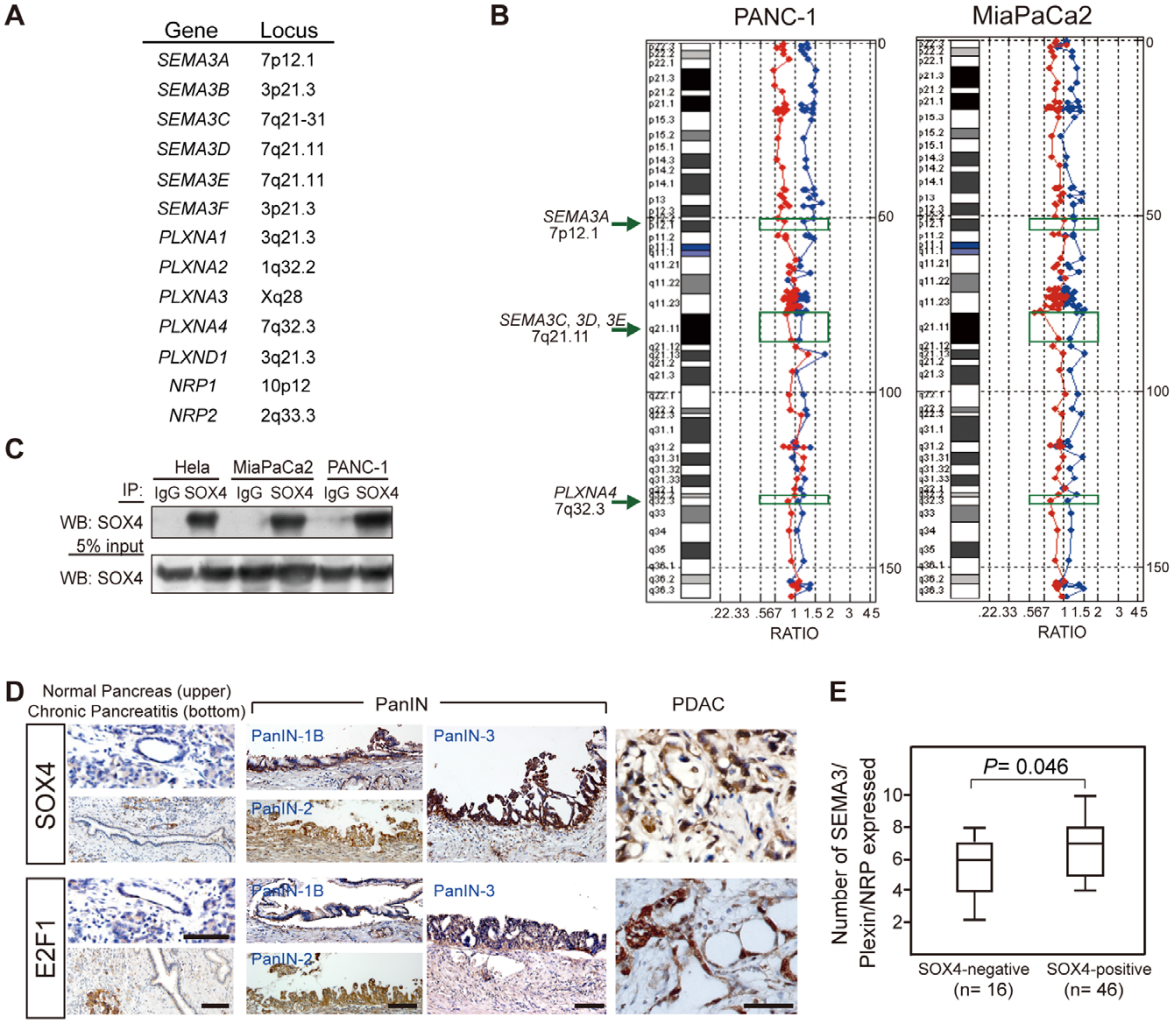
SOX4 expression in PDAC correlates with the total number of expression of SEMA3/Plexin/NRP1. (A) gene loci of each human *SEMA3*, *Plexin*, and *NRP* gene. (B) representative array CGH on chromosome 7 with genomic DNA extracted from PANC-1 and MiaPaCa2 cells, respectively, compared with normal human genomic DNA (blue dots and line: forward experiment, cell line versus control; red dots and line: reverse experiment, control versus cell line). No significant gene amplification (> =  or < =  2-fold change) was observed around *SEMA3A*, *3C*, *3D*, *3E* and *PLXNA4* gene loci (indicated by green-colored rectangle and arrow). (C) SOX4 is expressed in PDAC cell lines. Cell lysates were immunoprecipitated using anti-SOX4 antibody and immunoblotted with SOX4 or a control IgG antibody. (D) immunohistochemistry of SOX4 and E2F1 in human PDAC cases. SOX4 and E2F1 were both detected in PanIN of different grades and invasive adenocarcinoma, but not in normal ductal and acinar epithelia or in chronic pancreatitis. Scale bar = 100 µm. (E) Rank sum of staining intensity for SEMA3/Plexin/NRP1 correlates with SOX4 expression in PDAC cases. The staining of each section was scored 1–3 to rank its intensity as weak, medium, or strong. Scores of all SEMA3/Plexin/NRP1 immunostaining for each case were summed and correlated with SOX4 expression (NPar Kruskal-Wallis one-way analysis). The median (line within the box), maximal and minimal values (upper and lower lines outside the box, respectively) for each group are shown in the boxplot.

Several mechanisms could underlie the observation that multiple members of the SEMA3 and Plexin family were co-upregulated in PDAC. First, genes encoding SEMA3 and Plexin family members may be co-amplified due to frequent copy number variations in pancreatic cancer. We have noticed that most genes of the human SEMA3 and Plexin families are located on either chromosome 3 or 7 (Fig. 2A). Consequently, we used array comparative genomic hybridization to explore the possibility of DNA amplification in SEMA3 and Plexin family gene loci. As shown in Fig. 2B, no change in DNA copy number was observed at chromosomal regions 7p12.1 (*SEMA3A* locus), 7q21 (*SEMA3C*, *SEMA3D*, *SEMA3E* loci) (Fig. 2B), 1q32.2 (*PLXNA2* locus), 3p21.3 (*SEMA3B* and *SEMA3F* loci), 3q21.3 (*PLXNA1* and *PLXND1* loci), or 10p12 (*NRP1* locus) (Supplementary Fig. S2A). Equivocal DNA amplification was observed at the chromosome Xq28 region only, where *PLXNA3* resides (Supplementary Fig. S2A).

Another common alteration in cancer cells is silencing of tumor suppressor genes by hypermethylation [8]. Although SEMA3F and SEMA3B are generally regarded as tumor suppressors and methylated *SEMA3F* has been reported in lung cancers [9], it is neo-expression instead of suppression of SEMA3/Plexin expression that is generally observed in PDACs (see Table 1). Further, CpG-rich islands for methylation modification are not always found in the predicted promoter regions of SEMA3/Plexin family genes (as predicted by the EMBOSS CpGPlot Program). As a result, we next investigated the possibility that co-expression of SEMA3/Plexin/NRP family members in PDACs is caused by the neo-expression of some master control gene(s) crucial for tumorigenesis of PDAC, possibly transcription factor(s) that could interact with a consensus DNA binding motif present in the promoter of each SEMA3/Plexin/NRP gene. Several candidates, including E2F1, GATA6, and SOX2, were obtained via a literature search. The E2F1 transcription factor could activate *NRP1* transcription in mouse cortex under ischemic change [10]. Mouse *SEMA3C* and *PLXNA2* are the direct targets of GATA6 during cardiac development [11]. Finally, *SEMA3C* and *NRP1* were reported to be the direct target genes of SOX4 [12]. Accordingly, we attempted to locate the SOX4-consensus binding site [(A/T)(T/A)CAA(A/T)G], the GATA6-binding site [(A/T/C)GAT(A/T)(A)], and the E2F1-binding site [TTT(C/G)(C/G)CGC] in predicted promoters for each SEMA3 and Plexin gene [12]–[15]. As listed in Supplementary Table S3, all SEMA3, Plexin, and NRP genes possess at least one putative SOX4 binding site and one E2F1 binding site in the predicted promoter regions.

The presence of the putative SOX4 and E2F1 binding sites in the promoter of each SEMA and Plexin gene prompted us to examine SOX4 and E2F1 expression in human PDAC tissues and cell lines. Immunoblotting showed SOX4 expression in PANC-1 and MiaPaCa2 cells (Fig. 2C). For human PDAC samples, immunohistochemistry revealed that 76.4% of human PDAC expressed SOX4, and 87.9% expressed E2F1 (Fig. 2D). By contrast, acinar and ductal epithelial cells from normal pancreas and chronic pancreatitis were not immunoreactive to SOX4 or E2F1. Moreover, SOX4 and E2F1 were already detected in PanIN of different grades (Fig. 2D), suggesting that SOX4 and E2F1 are de novo expressed in the early processes of PDAC tumorigenesis.

To explore the relationship between the expression of SOX4 or E2F1 and that of SEMA3/Plexin/NRP1 in patients with PDAC, we ranked the staining intensity of each SEMA3 and Plexin as a number from 0–3 (0, negative; 1, weak; 2; moderate; 3, strong) and analyzed each patient's rank sum versus SOX4 or E2F1 expression. *NPar* Kruskal-Wallis *one*-*way* analyses showed significant differences in the rank sum of SEMA3, Plexins and NRP1 staining between SOX4-positive and SOX4-negative cases (*P* = 0.046) (Fig. 2E). However, no difference was observed when E2F1 expression was considered as the variable (*P* = 0.9121). The highly correlated expression of SOX4 with SEMA3/Plexin/NRP1 family members in human PanIN and PDAC suggests that SOX4 might function as a master transcription factor to drive the expression of SEMA3 and Plexin genes in pancreatic cancer formation.

### SOX4 activates the transcription of SEMA3 and Plexin genes

**Figure 3:**
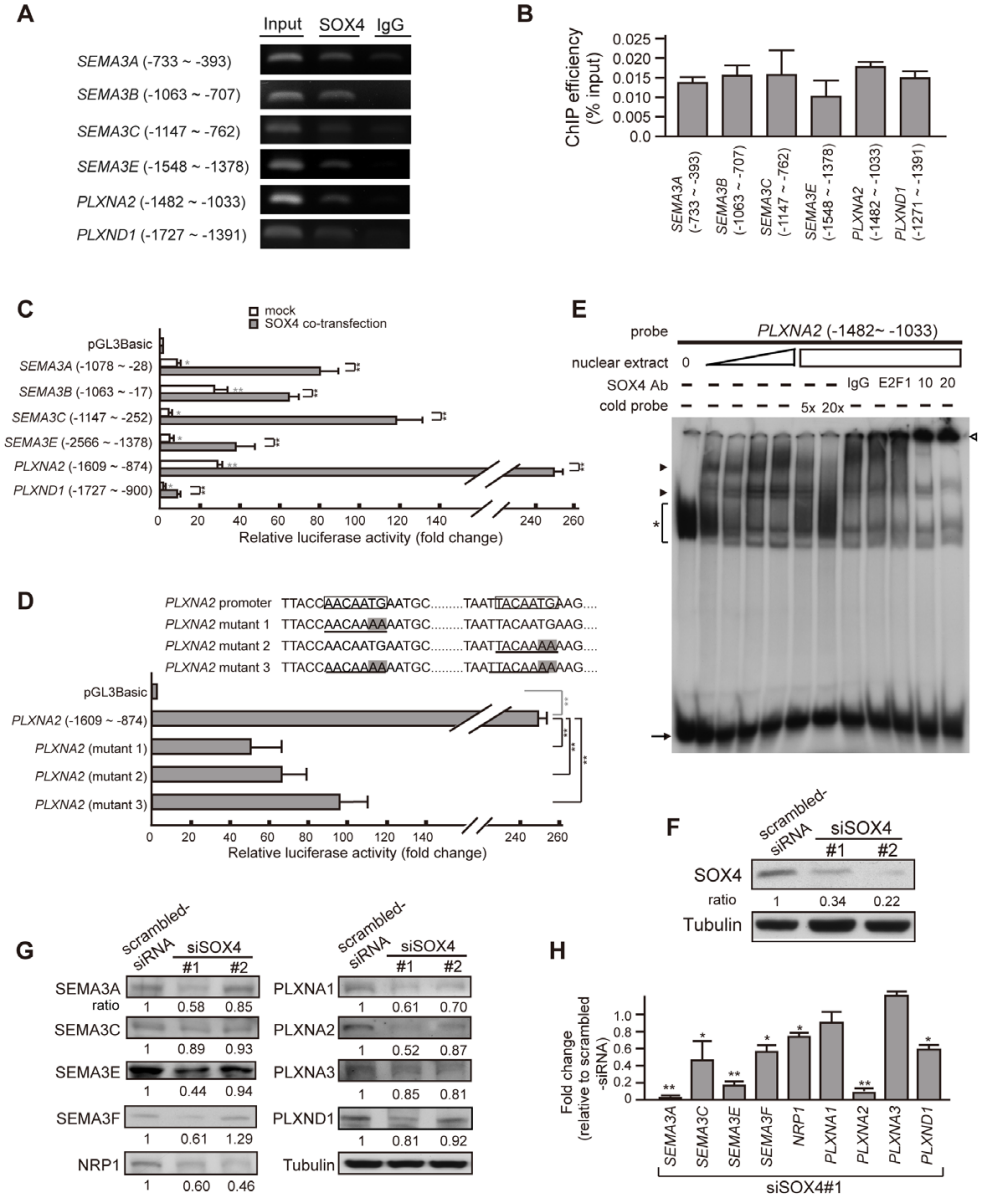
SOX4 trans-activates the transcription of *SEMA3*, *Plexin*, and *NRP1*. (A) representative end-point PCR of chromatin immunoprecipitation (ChIP) using an antibody against SOX4 in PANC-1 cells. Genomic DNAs were amplified with primer sets after bound proteins were digested with proteinase K. The ChIP results showed binding of SOX4 to the genomic region harboring SOX4 binding sites in the predicted promoter of most *SEMA3* and *Plexin* genes. (B) quantification of ChIP results in (A), n> = 3. Each lane in figure 3A was given a value measured by densitometry and normalized with the value of IgG. ChIP efficiency is defined as percentage of the value derived from PCR of SOX4-immunoprecipitate relative to that of total genomic input. *Error bars*, standard deviation (SD) from at least triplicate. (C) increased luciferase activity of constructs containing *SEMA3*- or *Plexin*- promoter in the absence (white bars) or presence (gray bars) of co-expressed SOX4 in comparison with the promoterless pGL3-Basic control. The constructs harboring a SOX4-consensus binding site were transiently transfected into SOX4-containing HeLa cells. Shown are ratios of firefly luciferase expression to Renilla luciferase expression (expressed from co-transfected plasmid), measured 48 h after transfection, normalized to the mean ratio from the pGL3-Basic. Error bars show SD, n> = 4. Student's *t*-test, *, *P*<0.01; **, *P*<0.001. In the presence of co-transfected SOX4, luciferase activity driven by each *SEMA3*- or *Plexin*- promoter is further augmented (gray bars) compared to mock-transfected (white bars) and controls. (D) Transcriptional activity of *PLXNA2* promoter driven by SOX4 is attenuated when the consensus SOX4-binding sites are mutated. Boxed DNA sequence, the consensus SOX4-binding site; Underline with shadow in letters, the mutated SOX4-binding sites. Bar graph shows that mutation of either SOX4-binding site caused significant decrease in luciferase activity driven by *PLXNA2* promoter in the presence of overexpressed SOX4. Error bars, SD, n = 5. Student's *t*-test, *, *P*<0.01; **, *P*<0.001. (E) EMSA shows that the migration of a radiolabeled DNA fragment derived from the *PLXNA2* promoter is retarded by SOX4-containing HeLa crude nuclear extract in a concentration-dependent manner. The signal of protein-bound retarded band (arrowheads) was specifically diminished by cold probes containing SOX4-binding consensus sequences and was super-shifted by anti-SOX4 antibody (empty arrowhead). Asterisk, non-specific binding signals; arrow, un-bound radiolabeled probe. (F) effective RNAi knockdown of endogenous SOX4 in PANC-1 cells shown by SOX4 immunoblotting. The value indicated is the normalized relative intensity measured by densitometry (The ratio SOX4/Tubulin for scrambled-siRNA cells is defined as 1.). Two stable siSOX4 clones labeled as siSOX4#1 and siSOX4#2 were constructed with the targeting oligonucleotide directed against different nucleotide sequences in *SOX4* gene (siSOX4#1, nt.1999–2019 NM_003107.2; siSOX4#2, nt.1362–1382 NM_003107.2). (G) Immunoblotting in SOX4-depleted cells shows a concomitant decrease in the protein amount of NRP1, SEMA3- and Plexin- family members. Tubulin was used as a standard for normalization in the quantification of each lane measured by densitometry. The ratio indicated was the normalized value in siSOX4 cells relative to the normalized value in scrambled-siRNA cells. (H) Real-time PCR to quantify mRNA expression of *SEMA3* and *PLXN* genes in SOX4-depleted cells relative to SOX4-abundant scrambled-siRNA cells (shown by fold-change). Student's *t*-test, *, *P*<0.01; **, *P*<0.001.

To demonstrate that SOX4 could regulate the transcription activity of SEMA3 and Plexin genes in PDAC, we first investigated whether SOX4 could bind to the predicted promoter of each SEMA3 and Plexin gene. Chromatin immunoprecipitation performed on PANC-1 cells showed that SOX4 indeed bound to the promoter of each SEMA3 and Plexin gene (Fig. 3A and 3B). Significant fold-increases in luciferase activity were observed in the construct driven by the promoter derived from SEMA3 and Plexin gene when transfected into SOX4-expressing HeLa cells. In addition, co-transfection of a SOX4-expressing vector further enhanced the promoter activity (Fig. 3C, P<0.001), which was attenuated when the SOX4-consensus binding sites were mutated (AACAATG to AACAAAA and TACAATG to TACAAAA mutations) (Fig. 3D). Similarly, electrophoresis mobility shift assay showed that protein extracted from SOX4-containing nuclei retarded the migration of a radio-labeled DNA probe composed of *SEMA3* or *Plexin* promoter sequences in a concentration-dependent manner. This effect was specifically relieved by a cold-labeled probe composed of SOX4-consensus binding sequences and could be supershifted by the addition of SOX4 antibodies (Fig. 3E). Conversely, RNAi-mediated depletion of SOX4 in PANC1 cells (66% and 78% reduction in SOX4 protein for siSOX4#1 and siSOX4#2, respectively, Fig. 3F) resulted in a concomitant decrease in the mRNA and protein levels of most SEMA3/Plexin family members and NRP1 (Fig. 3G and 3H). Collectively, these results suggest that SOX4 could function as a master factor to activate the transcription of SEMA3 and Plexin genes simultaneously, leading to the expression of multiple SEMA3 and Plexin family members in pancreatic cancer cells.

### RNAi-mediated depletion of SOX4 suppresses tumor growth *in vitro* and *in vivo*

**Figure 4:**
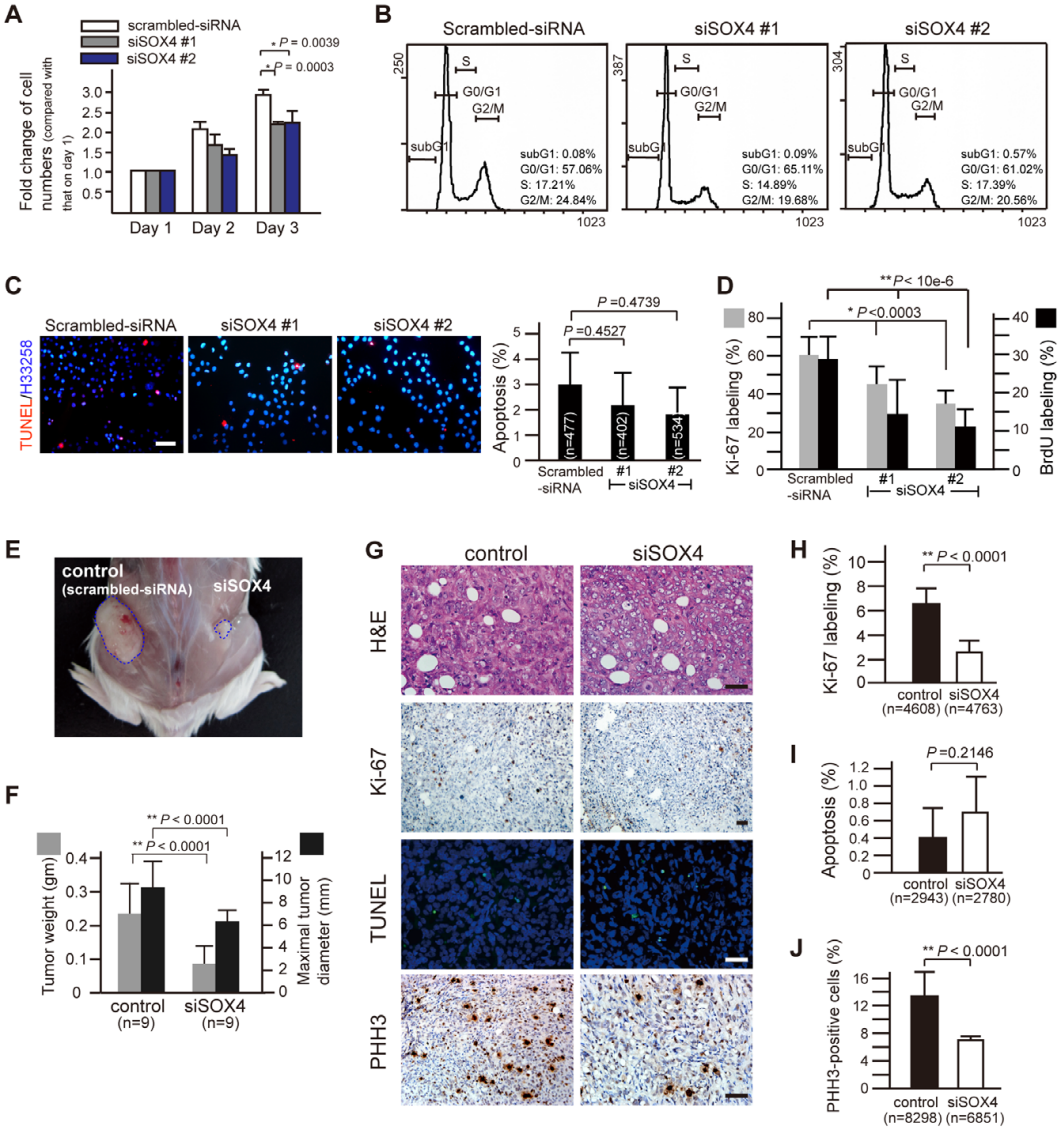
Reduced *in vitro* and *in vivo* tumor growth by SOX4 suppression. (A) Cell proliferation is affected in PANC-1 cells with RNAi-suppression of SOX4. 30000 cells were seeded into each well in a 12-well plate. At 24, 48 or 72 hours after seeding, cells were harvested and counted by trypan blue exclusion assay. *Error bars*, SD from six independent experiments, Student's *t*-test. (B) Representative flow cytometry of siSOX4 cells stained with propidium iodide shows no significant variation from scrambled-siRNA cells in apoptosis (depicted by sub-G1) or in cell cycle progression. The percentage of cells in sub-G1, G0/G1, S and G2/M phase was shown at the right downward corner of each plot. (C) representative TUNEL-labeling of cells challenged by the genotoxic agent cisplatin (10 µg/ml, 12-h). Differences in apoptosis was not observed between SOX4-knockdown (siSOX4#1 and siSOX4#2) and control (scrambled-siRNA) cells. (D) lower cell proliferation rate and decreased number of cells staying at M phase in siSOX4 cells. siSOX4 and scrambled-siRNA cells were plated on coverslips and incubated for 48 hours. Before harvest and fixation with paraformaldehyde, cells were incubated with 10 µM of BrdU for 3 hours and were then subjected to anti-Ki-67 or anti-BrdU immunofluorescent staining. Twenty randomly selected high power fields (x400) were counted for statistical analysis (Student's *t*-test). (E) Tumor xenograft of siSOX4/PANC-1 cells grows smaller than that of control cells (scrambled-siRNA) in a SCID mouse. The mice were sacrificed 30 days after subcutaneous inoculation of the control cells on the left and the siSOX4 cells on the right side of the flank. (F) Smaller and lighter tumor xenografts of siSOX4 cells compared to those of control (n = 9, Student's *t*-test). (G) representative H&E, Ki-67, PHH3 immunostain, and TUNEL labeling in tissue sections from tumor xenografts. Scale bars = 50 µm. (H, I, and J) quantification of proliferation (Ki-67 labeling), apoptosis (TUNEL stain) and mitosis (PHH3-immunostain). Eighteen randomly selected fields (x200) in each Ki-67-stained section and cells in twenty-seven randomly selected fields (x400) for TUNEL labeling or PHH3 immunostain were counted in each xenograft tumor. The proliferating index and the mitotic counts of siSOX4 tumor cells are significantly lower than that of control cells, while the percentage of apoptotic cells did not differ (Student's *t*-test). n, the total number of cells counted.

To investigate how SOX4 affects tumorigenesis in PDAC cells, the effect of SOX4 knockdown on cell proliferation and cell cycle progression was first examined. It showed that the total number of viable cells was significantly decreased in SOX4-knockdown cells cultured *in vitro* for three days (Fig. 4A). Cell cycle progression and apoptosis were quantitatively compared between SOX4-suppressed and -competent cells, and no significant difference was observed by flow cytometry or TUNEL assay challenged by genotoxic agent (Fig. 4B and 4C). In contrast, proliferation of SOX4-suppressed (siSOX4 #1 and siSOX4 #2) cells was obviously decreased, as less Ki-67 immunoreactivity and much less BrdU labeling were observed in siSOX4 cells (Fig. 4D), indicating that the number of cells entering the cell cycle is reduced when SOX4 is depleted. This finding suggests that SOX4 might function to facilitate cell proliferation when expressed in pancreatic cancer cells.

Using an *in vivo* tumorigenesis assay, we also demonstrated that tumors growing from SOX4-suppressed cells were much smaller in size and weight compared with those growing from control SOX4-competent cells (Fig. 4E and 4F). This *in vivo* growth retardation of tumors conferred by SOX4 depletion was generally attributed to decreased cell proliferation and proportionally decreased numbers of cells staying in the M phase, as shown by less Ki-67- and phosphohistone-H3 (PHH3)-immunoreactivity (Fig. 4G, 4H, 4J), but no increase in apoptosis was noted via TUNEL assay (Fig. 4G and 4I). Collectively, these data indicate that SOX4 could mediate tumor growth via regulation of cell proliferation in pancreatic cancer.

### Expression of SOX4 in pancreatic cancers correlates with poor survival, and is strongly associated with co-repressed expression of microRNA-129-2 and microRNA-335

**Figure 5:**
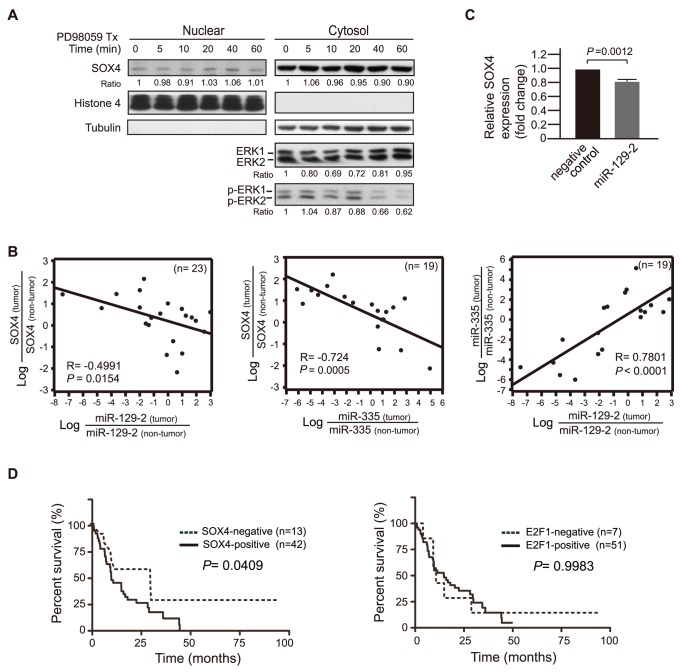
Co-repressed miR-129-2 and miR-335 are associated with expression of SOX4, which correlates with shorter survival in patients with pancreatic cancer. (A) time-course change of ERK1/2 phosphorylation and the cytoplasm-nucleus distribution of SOX4 after PD98059 treatment. The whole cell lysates at the indicated time after treatment with 20 µM PD98059 were subjected fractionation, resolved by SDS-PAGE, and immunoblotted with the indicated antibodies. At 40 min after PD98059 treatment when ERK1/2 phosphorylation was inhibited, no difference was observed in the total amount of SOX4 protein, or the cytoplasm-nuclear distribution of SOX4. (B) *Left* and *Central* plots: Strong inverse correlation between miR-129-2 expression/miR-335 expression and SOX4 expression in PDAC cases as analyzed by Pearson correlation with log transformation for normality in scatter plots. n, the number of cases analyzed; Negative value in R indicates inverse correlation between X- and Y-variables. *Right* plot: the expression level of miR-335 in pancreatic carcinoma samples is positively correlated with the level of miR-129-2 in a linear regression way. (C) Marginal effect on suppressing SOX4 expression by transient transfection of miR-129-2 in PANC-1 cells as assessed by quantitative real-time PCR analysis in triplicate using a paired *t*-test. (D) Kaplan-Meier curves were shown as a function of SOX4 or E2F1 immunohistochemistry. *Left*: SOX4 expression in human PDAC correlates with shorter patient survival. *Right*: E2F1 expression did not correlate with patient survival. The *P*-value corresponds to the log-rank test by comparing the survival curves.

More than 80∼90% of pancreatic cancers harbor mutations in codon 12 of *KRAS* resulting in constitutive activation of the K-RAS/B-RAF/MAPK signaling pathway [16]. Similarly, we found that 80% of human PDAC cases collected in this study harbor an activating mutation in *KRAS* at codon G12 (Supplementary Fig. S4A and S4B). Thus, we asked whether a hyperactivated RAS/MEK/ERK signal is responsible for SOX4 over-expression in pancreatic cancer. As shown in Fig. 5A, phosphorylated ERK1/2 and nuclear SOX4 were both detected in PANC-1 cells. Inhibition of MEK/ERK activity by PD98059 in pancreatic cancer cells had no effect on the SOX4 protein level or the status of SOX4 nuclear localization. As a result, we suggest that RAS/MEK/ERK signaling is not involved in the regulation of SOX4 expression in pancreatic cancer cells.

We also examined the possibility of a *SOX4* gene mutation or amplification that might account for SOX4 over-expression. Direct genomic DNA sequencing of the *SOX4* gene revealed a normal genotype (Supplementary Fig. S3A), and comparative CGH over *SOX4* loci (6p22.3) showed no obvious gain or loss (Supplementary Fig. S3B). We then turned to explore whether mis-regulation of microRNAs might be responsible for changes in SOX4. The SOX4 transcript is a target of both miR-129-2 and miR-335. Elevation of SOX4 expression associated with repressed miR-129-2 or miR-335 is reported in gastric-, endometrial-, and breast cancers [17]–[19]. Accordingly, we examined the expression levels of miR-129-2, miR-335, and SOX4 in twenty-three paired human pancreatic tumors and normal tissues. The level of miR-129-2 or miR-335 was decreased in most pancreatic carcinoma samples compared to that of tumor-uninvolved tissues in the same patient and was associated with concomitant increased mRNA levels of SOX4 (Fig. 5B). Pearson correlation analysis further demonstrated that the relative expression level of SOX4 in pancreatic carcinomas (normalized with the value derived from non-tumor tissues) was inversely correlated with that of miR-129-2 and miR-335, respectively, in a statistically significant way (Pearson correlation coefficient R = −0.4991, *P* = 0.0154 for LogmiR-129-2 vs logSOX4; R = −0.724, *P* = 0.0005 for logmiR-335 vs logSOX4) (Fig. 5B, left and central plot). Moreover, the repressed expression of miR-335 in pancreatic carcinoma samples was positively correlated with the decreased level of miR-129-2 (Fig. 5B, right plot), indicating a concomitant repression of miR-129-2 and miR-335 in pancreatic cancers. Intriguingly, re-introduction of miR-129-2 into PDAC cell lines had mild suppressive effect on the level of SOX4 mRNA (Fig. 5C), suggesting functional redundancy with other microRNAs such as miR-335. Finally, to elucidate the biological significance of SOX4 in pancreatic cancers, Kaplan-Meier survival analysis was performed to demonstrate that SOX4 expression in tissues from human PDAC significantly correlates with shorter survival of patients (*P* = 0.0409). In contrast, no statistical correlation is observed between E2F1 expression and patients' survival despite strong expression of E2F1 in PDAC (Fig. 5D).

## Discussion

Mounting evidence indicates that axon-guidance molecule groups such as Semaphorins, Eph, and netrins play a role in tumorigenesis and tumor progression. During embryonic development and cancer formation, the axon guidance molecules function by inducing cytoskeleton changes to affect cell motility, regulating cell proliferation, apoptosis, angiogenesis, or altering immune responses [4], [20]–[22]. Members of the class 3 Semaphorin and Plexin families have been individually reported to be functionally involved in tumorigenesis or tumor progression in different types of cancers. However, concurrent expression of multiple class 3 Semaphorins associated with their cognate receptors, Plexin and NRP1 has never been reported. We first reported that more than five members of SEMA3/Plexin family are expressed in pancreatic cancer and its precancerous lesion. Given that RNAi-knockdown of a single SEMA3 or Plexin results in no significant effect on tumor growth and that multiple RNAi-knockdown of SEMA3/Plexin is not experimentally feasible, the biological significance underlying concomitant expression of multiple SEMA3/Plexin family members in pancreatic cancer remains incompletely understood. It may reflect only a “passenger mutation” phenomenon, as the cancer genome is inherently unstable [23]. Alternatively, the function of SEMA3 family members is redundant and requires participation of multiple SEMA3/Plexin members in concert to promote tumor development as suggested previously [24].

Dysregulation of gene expression in cancer cells could result from a variety of mechanisms, including alteration in genomic structure, aberrant transcriptional/post-transcriptional regulation, or dysregulated epigenomic modification [25]. Our study reveals that the concomitant expression of SEMA3 and Plexin family members is not due to gene amplification but can be partially attributed to increased transcriptional activity driven by an SRY-HMG Box transcription factor SOX4 de novo expressed in pancreatic cancer. SOX4 controls multiple developmental events and is widely conserved across genomes. During mammalian embryonic development, SOX4 regulates multiple cell types including chondrocytes, osteoblasts, thymocytes, semilunar valve cells, and muscular ventricular septum cells of the cardiac outflow tract [26]–[29]. Intriguingly, SOX4 regulates neuronal differentiation in the developing nervous system by augmenting transcription of genes including GnRH, PCDHB, MYB, RBP1, and TEAD2 [30]–[32]. In this study, we demonstrate that SOX4 can trans-activate many Semaphorin and Plexin genes, which together compose the largest family of axon guidance molecules. Our findings further support a general modulating role of SOX4 in the developing nervous system as a transcription regulator by targeting various genes involved in neural development.

SOX4 can also regulate tumorigenesis of different tissue types by acting as a driver oncogene. SOX4 is widely expressed in various types of cancer including glioblastoma, medulloblastoma, melanoma, and adenoid cystic carcinoma of salivary gland, as well as colon-, prostate-, bladder- and lung cancers [13], [14], [33]–[40]. Here, we have reported the neo-expression of SOX4 in both pancreatic precursor lesions and defined cancer. Intriguingly, expression studies of SOX4 have demonstrated that SOX4 is initially confined to the pancreatic epithelium at E12.5 in mouse embryos and later shifts to islets at E18.5, suggesting SOX4 involvement in pancreatic development [41]. It seems reasonable for pancreatic cancer cells to re-express SOX4 considering that tumor cells often aberrantly re-express developmentally regulated genes for advantageous growth or cell motility.

The pro-oncogenic function of SOX4 in pancreatic cancer is relevant to the control of cell proliferation as evidenced by decreased cell proliferation rates in SOX4-suppressed cells. Although SOX4 is reported to function as a survival factor during the development of neuro-progenitors [42] and SOX4 mediates prostaglandin A2-induced apoptosis in human hepatocellular carcinoma [43], we did not detect a significant effect on apoptosis with SOX4 depletion in pancreatic cancer cells. It is always possible that incomplete depletion of SOX4 by RNA interference might account for no detectable effect on apoptosis conferred by siSOX4. However, given that the apoptotic activity of SOX4 is mediated through a glycine-rich central domain that is unrelated to its transcriptional trans-activation activity [44], it is likely that SOX4 performs a unique biological effect in different cellular contexts depending on differential protein-protein interaction with each SOX4 protein motif/domain. Intriguingly, SOX4 can interact with TGF-β, Wnt, Notch and PI3K signaling pathways and activates β-catenin to promote tumor proliferation [45]. In glioblastoma, SOX4 serves as a direct TGF-β target gene, conducts an alternative non-SMAD TGF-β signaling, and induces SOX2 expression to maintain the stemness of glioma-initiating cells [34], [46]. Therefore, it remains to be investigated whether SOX4 interacts with the non-SMAD TGF-β signaling to regulate β-catenin activity for promotion of cell proliferation in pancreatic cancer.

By which mechanism could SOX4 be expressed during PDAC tumorigenesis? Over-expression of SOX4 in human non-small cell carcinoma of lung and urothelial tumor has been reported to result from gene amplification [39], [47]. However, comparative genomic hybridization analyses of pancreatic cancer (by this lab and others) did not reveal a significant copy number change of the *SOX4* locus (6p22.3). This finding suggests that there is less of a possibility of a genomic copy number gain such as that which underlies the over-expression of several other molecules, including *c-ERBB-2*, *Cyclin-D1*, *AURKA*, and *GATA6* in pancreatic cancer [48], [49]. Detailed genetic makeup studies indicate that human pancreatic cancer contains an average of 63 genetic alterations, most of which are point mutations [2]. Accordingly, we have performed a genomic DNA sequence analysis of the *SOX4* gene and found no evidence to support an activating mutation of the *SOX4* gene in human pancreatic cancers [39]. Instead, our study suggests one possible epigenetic modification mechanism for SOX4 expression in pancreatic cancer via repressed expression level of miR-129-2 and miR-335, which would otherwise target SOX4 transcript at its 3′ UTR for degradation [17], [50]. We clearly demonstrate that the level of miR-129-2 expression or of miR-335 is inversely correlated with SOX4 expression in most patients with pancreatic cancer in a statistically significant way. The elevation of SOX4 has been reported to be a consequence of miR-129-2 repression in endometrial, gastric, and bladder cancer [17], [18], [51], [52], or as a consequence of miR-335 repression in metastatic breast cancer [50], suggesting the miR-129-2/SOX4 or miR-335/SOX4 regulatory axis as a general strategy for tumor formation or progression. However, *in vitro* introduction of miR-129-2 into PANC cells is insufficient to achieve significant physiological effects, indicating redundancies may exist between miRNA seed sequences and target mRNA. Given the multiple genetic alterations that are present in pancreatic cancer and the minimal physiological effect conferred by the re-expression of miR-129-2 in pancreatic cancer cell lines *in vitro*, more than one mechanism may contribute to over-expression of SOX4 in pancreatic cancers. It would be interesting to investigate whether collaborative interaction between suppressed miR-129-2 and suppressed miR-335 in pancreatic epithelial cells could achieve robust induction of SOX4 in initiating or maintaining PDAC tumorigenesis. An alternative could be other epigenetic modifications such as hypomethylation of the SOX4 gene, as exemplified by hypomethylated *Claudin4*, *Lipocalin2*, and *S100A4*, which accounts for their overexpression in pancreatic cancers [53]. It would also be interesting to explore the mechanism for repressed miR-129-2 in pancreatic cancer, in light of the finding that hypermethylation of its miRNA promoter CpG island accounts for miR-129-2 suppression in endometrial cancer [17].

## Supporting Information

Methods S1 **Primers for real-time RT-PCR.**
(DOC)

Figure S1 **Kaplan-Meier curves for overall survival correlated with the expression level of each SEMA3 or Plexin.** Samples were grouped according to the expression level of each individual member of SEMA3 or Plexin family assessed by immunohistochemistry. The *P* value was estimated from the log-rank test.
(TIF)

Figure S2 **(A) array CGH with genomic DNA extracted from PANC-1 and MiaPaCa2 cells in comparison with normal human genomic DNA.** No significant gene amplification (> =  or < =  2-fold change, green rectangle) was observed around *SEMA3B*, *SEMA3F*, *PLXNA1-3*, *PLXND1* or *NRP1* loci. (B) repression of PLXNA2 expression via RNA interference in PANC-1 cells as shown by immunoblotting. The amount of PLXNA2 protein was normalized to endogenous tubulin and expressed as a ratio. (C) no difference in cell cycle progression between PLXNA2-knockdown (siPLXNA2) and control cells by flow cytometry. (D) Cell proliferation is not affected in PANC-1 cells with RNAi-suppression of PLXNA2. *Symbols* on lines, mean cell numbers at each indicated time point. *Bars*, SD from triplicates. (E) Electrophoresis mobility shift assay shows that the migration of radiolabeled DNA fragment derived from *NRP1* promoter is retarded by SOX4-containing HeLa crude nuclear extract in a concentration-dependent manner. Signal from the protein-bound retarded band (arrowheads) is specifically diminished by cold probes containing SOX4-binding consensus sequences.
(TIF)

Figure S3 ***SOX4*** **gene is not mutated or amplified in human pancreatic cancer samples and cell lines.** (A) representative genomic sequencing results derived from one human pancreatic cancer tissue showed no alteration (indicated by gray arrows in each panel) at the indicated mutational hotspots of *SOX4* gene (expressed as gray-colored words). (B) array CGH result with genomic DNA extracted from PANC-1 and MiaPaCa2 cells showing no significant gene amplification around the SOX4 locus at chromosome 6p22 (> =  or < =  2-fold change, green rectangle).
(TIF)

Figure S4 **(A) A summary table listing mutational analysis at** ***KRAS*** **codon 12 in forty-four pancreatic cancers collected in this study.** Eighty percent of PDAC samples have activating mutation in *KRAS* at codon 12 including G12D, G12R and G12V mutation. (B) representative genomic sequencing results derived from human pancreatic cancer tissues showed point mutation at the indicated mutational hotspots of *KRAS* codon 12.
(TIF)

Table S1 **Demographic characteristics of patients with PDAC (n = 62).**
(DOC)

Table S2 **Statistical analysis for tumorous nodal metastasis in correlation with the expression of each SEMA3, Plexin, and Neuropilin.**
(DOC)

Table S3 **Putative SOX4 and E2F1 binding sites (position from the transcription starting sites).**
(DOC)
