# Supplementary material for: SOX4 Transcriptionally Regulates Multiple SEMA3/Plexin Family Members and Promotes Tumor Growth in Pancreatic Cancer
Source: PLoS One. 2012 Dec 12;7(12):e48637. doi: 10.1371/journal.pone.0048637 (PMC3520963; doi:10.1371/journal.pone.0048637)
Supplement: Methods S1 — Primers for real-time RT-PCR. (DOC) [file pone.0048637.s001.doc]

**Supplementary Materials and methods**

***Primers for real-time RT-PCR***

Primers used for microRNAs are: *miRNA-129-2-3p*, 5'-AAGCCCTTACCCCAAA-3' and 5'-TGGCAGGGTCCGAGGT-3'; *miRNA-129-2-5p*, 5'-CTTTTTGCGGTCTGGG-3' and 5'-TGGCAGGGTCCGAGGT-3'; *miRNA-335,* 5'-TCAAGAGCAATAACGAAAAATGT-3' and 5'-GCTGTCAACGATAGGCTACGT-3'; *U6*: 5'-GTGCTCGCTTCGGCAGCACA-3' and 5'-TGGCAGGGTCCGAGGT-3'.

***Primers for end-point PCR after ChIP***

Primers used to amplify promoter sequences after ChIP are as follow. *SEMA3A* (NG_011489): 5'-AGGACCTATATTTGTCTTGAAC-3' and 5'-AGGCAAGCGTTGTTTT-3'; *SEMA3B* (NC_000003.11): 5'-TATTGGAGTCCACGGGT-3' and 5'-CTCTGACGGCTCCTTT-3'; *SEMA3C* (AC_000139.1): 5'-CACTCACCTCTTCCTCCTAC-3' and 5'-CCATAACCACACATCTACAG-3'; *SEMA3E* (NC_000007.13): 5'-GTGTAGGCACCACATTTACTTTTCC-3' and 5'-GCCAGTCTGATGAGCTACAGACC-3'; *PLXNA2* (NC_000001.10): 5'-ACAGGTATGGATCAGGC-3' and 5'-AGCGCACACGAGTTTA-3'; *PLXND1* (NC_000003.11): 5'-GATGGGTTACCAATAGCG-3' and 5'-CACAACCGATAGCCAG-3'.

***Primers used in the PCR reaction for detection of KRAS and SOX4 gene mutation***

The primers used to amplify exon 2 of KRAS gene are *KRAS*-forward: 5'-GAATGGTCCTGCACCAGTAA-3' and *KRAS*-reverse: 5'-GTGTGACATGTTCTAATATAGTCA-3'. Two pairs of primers used to amplify SOX4 are *SOX4*-forward -1: 5'-GTCTTCCCGTTCGGCGTGT-3', *SOX4*-reverse-1: 5'-CGTTGCCGGACTTCACCTTC-3'; *SOX4*-forward-2: 5'-TCCCTTTCATTCGAGAGGCG-3', and *SOX4*-reverse-2: 5'-GCTCACCTCGGGCGTGCAGTAG-3'. Amplified PCR products were gel eluted and subjected to automated sequencing (ABI 377 DNA Sequencer, Applied Biosystems).

***Transfection of miR-129-2 miRNA precursor***

Pre-miR™ miR-129-2 precursor molecules and Pre-miR™ negative controls (Ambion) were commercially purchased and transfected into PANC-1 cells as the manufacturer’s instruction. Forty-eight hours after transfection, cells were harvested for RNA extraction. The extracted RNAs were reverse-transcribed with SOX4-specific primers. Relative SOX4 mRNA expression levels were determined by quantitative real-time PCR.
