# Supplementary figures and images for: SOX4 Transcriptionally Regulates Multiple SEMA3/Plexin Family Members and Promotes Tumor Growth in Pancreatic Cancer
Source: PLoS One. 2012 Dec 12;7(12):e48637. doi: 10.1371/journal.pone.0048637 (PMC3520963; doi:10.1371/journal.pone.0048637)

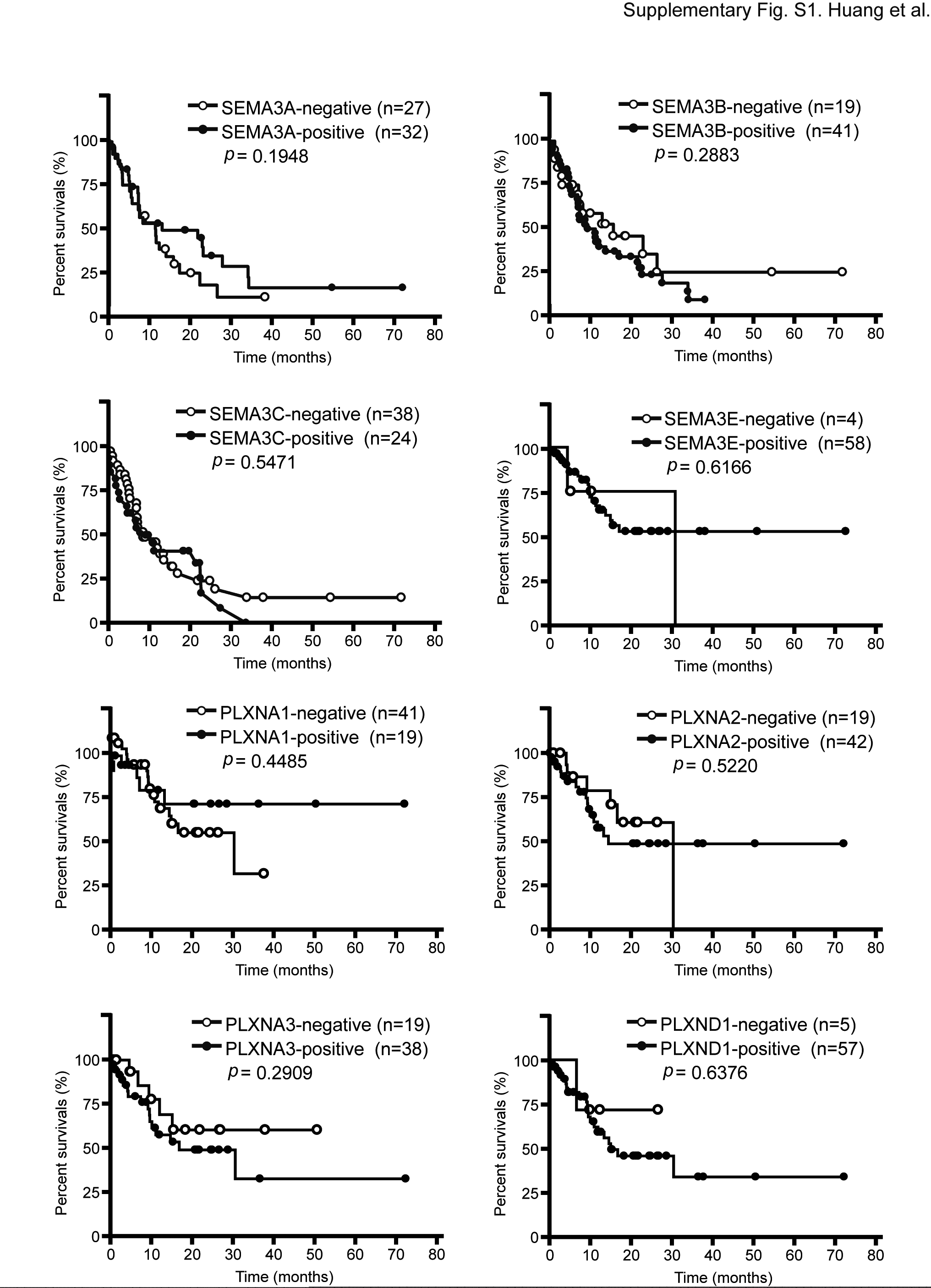

Supplement: Figure S1 — Kaplan-Meier curves for overall survival correlated with the expression level of each SEMA3 or Plexin. Samples were grouped according to the expression level of each individual member of SEMA3 or Plexin family assessed by immunohistochemistry. The P value was estimated from the log-rank test. (TIF) [file pone.0048637.s002.tif]

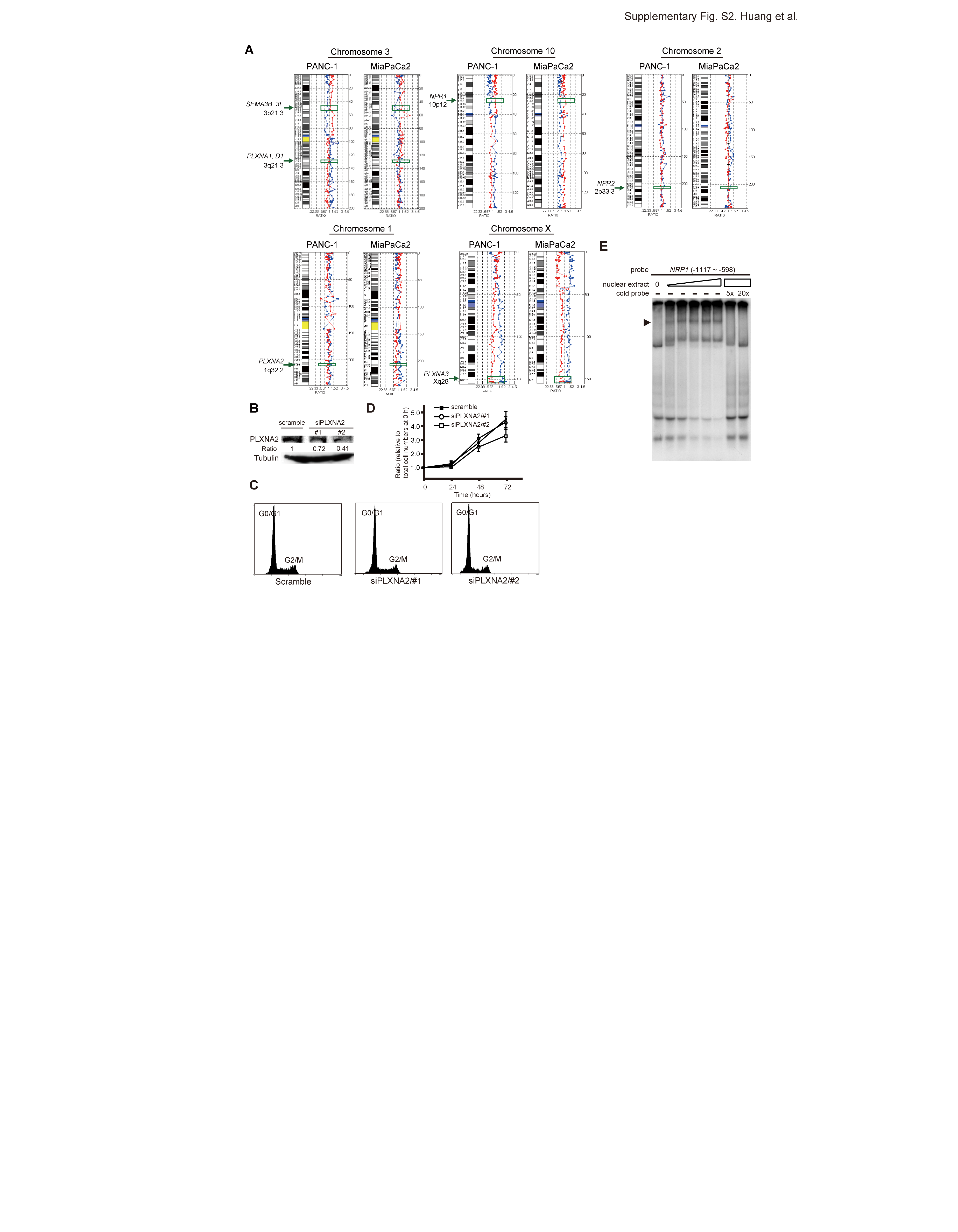

Supplement: Figure S2 — (A) array CGH with genomic DNA extracted from PANC-1 and MiaPaCa2 cells in comparison with normal human genomic DNA. No significant gene amplification (> = or < = 2-fold change, green rectangle) was observed around SEMA3B, SEMA3F, PLXNA1-3, PLXND1 or NRP1 loci. (B) repression of PLXNA2 expression via RNA interference in PANC-1 cells as shown by immunoblotting. The amount of PLXNA2 protein was normalized to endogenous tubulin and expressed as a ratio. (C) no difference in cell cycle progression between PLXNA2-knockdown (siPLXNA2) and control cells by flow cytometry. (D) Cell proliferation is not affected in PANC-1 cells with RNAi-suppression of PLXNA2. Symbols on lines, mean cell numbers at each indicated time point. Bars, SD from triplicates. (E) Electrophoresis mobility shift assay shows that the migration of radiolabeled DNA fragment derived from NRP1 promoter is retarded by SOX4-containing HeLa crude nuclear extract in a concentration-dependent manner. Signal from the protein-bound retarded band (arrowheads) is specifically diminished by cold probes containing SOX4-binding consensus sequences. (TIF) [file pone.0048637.s003.tif]

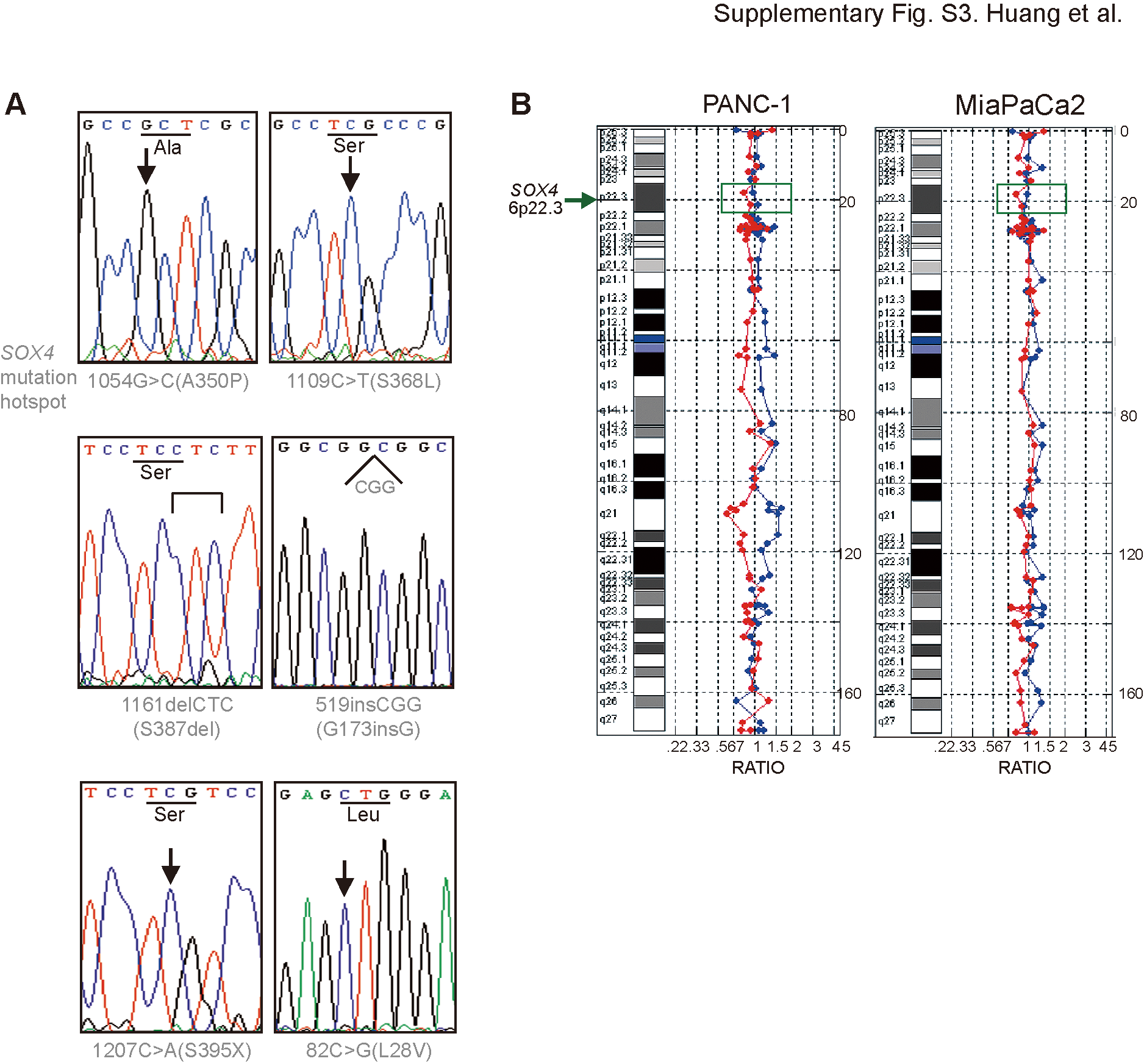

Supplement: Figure S3 — SOX4 gene is not mutated or amplified in human pancreatic cancer samples and cell lines. (A) representative genomic sequencing results derived from one human pancreatic cancer tissue showed no alteration (indicated by gray arrows in each panel) at the indicated mutational hotspots of SOX4 gene (expressed as gray-colored words). (B) array CGH result with genomic DNA extracted from PANC-1 and MiaPaCa2 cells showing no significant gene amplification around the SOX4 locus at chromosome 6p22 (> = or < = 2-fold change, green rectangle). (TIF) [file pone.0048637.s004.tif]

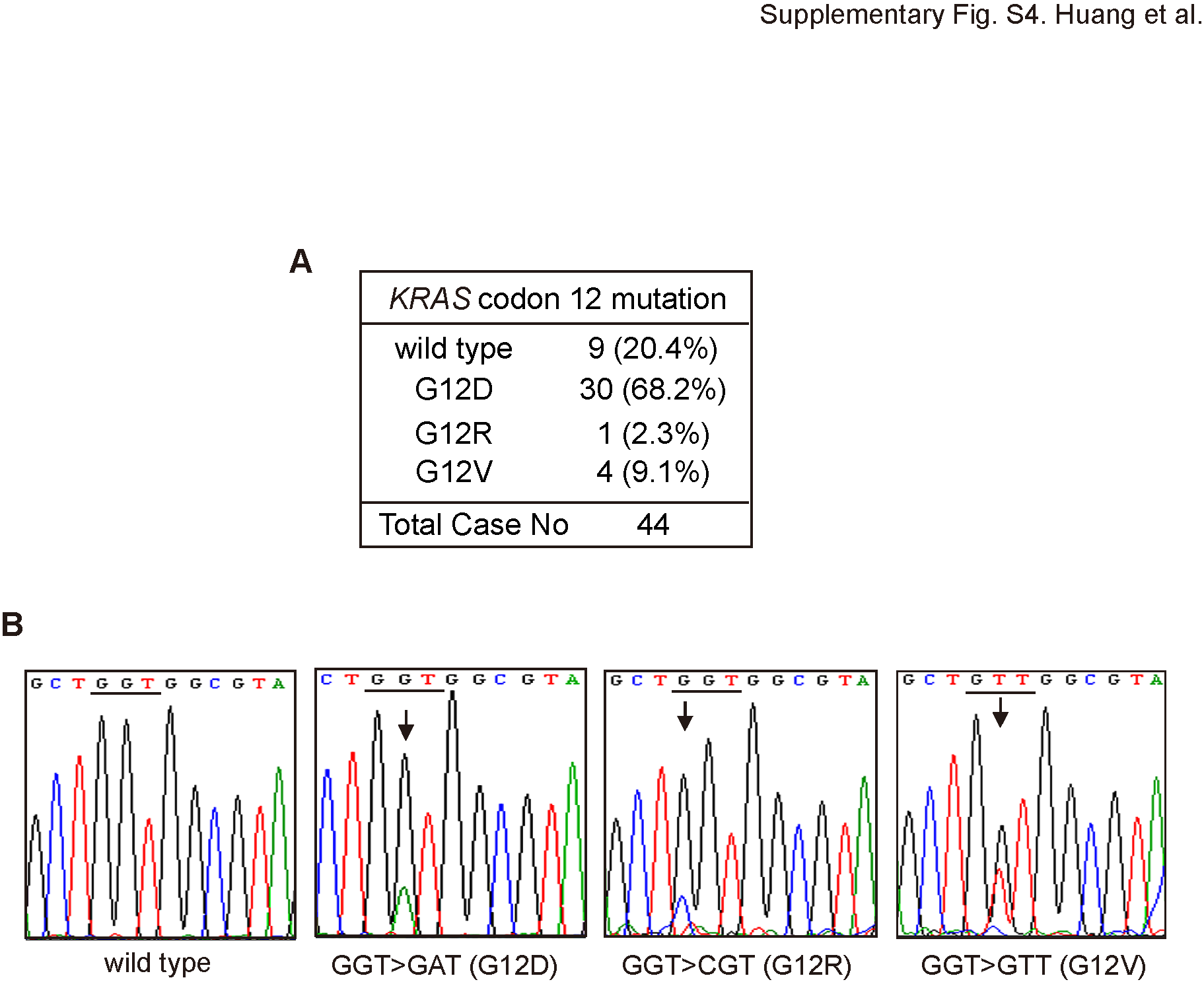

Supplement: Figure S4 — (A) A summary table listing mutational analysis at KRAS codon 12 in forty-four pancreatic cancers collected in this study. Eighty percent of PDAC samples have activating mutation in KRAS at codon 12 including G12D, G12R and G12V mutation. (B) representative genomic sequencing results derived from human pancreatic cancer tissues showed point mutation at the indicated mutational hotspots of KRAS codon 12. (TIF) [file pone.0048637.s005.tif]
