# Supplementary material for: SOX4 Transcriptionally Regulates Multiple SEMA3/Plexin Family Members and Promotes Tumor Growth in Pancreatic Cancer
Source: PLoS One. 2012 Dec 12;7(12):e48637. doi: 10.1371/journal.pone.0048637 (PMC3520963; doi:10.1371/journal.pone.0048637)
Supplement: Table S1 — Demographic characteristics of patients with PDAC (n = 62). (DOC) [file pone.0048637.s006.doc]

**Supplementary Table S1:**

Demographic characteristics of patients with PDAC (n=62)

| **Age (y/o)** | **30-95 (mean=64)** | | | |
| --- | --- | --- | --- | --- |
| **Gender** | **Male** | | **Female** | |
|  | **40** | | **22** | |
| **Histological grade**  **(differentiation)** | **Well** | **Moderate** | | **Poor** |
|  | **9** | **50** | | **3** |
| **Nodal metastasis** | **Absent** | **Present** | | **Unknown** |
|  | **24** | **31** | | **7** |
| **Survival (months)** | **Alive (n=17)** | | **Death (n=45)** | |
|  | **1.0 ~ 93.6 (mean=26.8)** | | **0.8 ~ 44.17 (mean=13.3)** | |
