# Supplementary material for: SOX4 Transcriptionally Regulates Multiple SEMA3/Plexin Family Members and Promotes Tumor Growth in Pancreatic Cancer
Source: PLoS One. 2012 Dec 12;7(12):e48637. doi: 10.1371/journal.pone.0048637 (PMC3520963; doi:10.1371/journal.pone.0048637)
Supplement: Table S2 — Statistical analysis for tumorous nodal metastasis in correlation with the expression of each SEMA3, Plexin, and Neuropilin. (DOC) [file pone.0048637.s007.doc]

**Supplementary Table S2: Statistical analysis for tumorous nodal metastasis in correlation with the expression of each SEMA3, Plexin, and N**europilin

| **Molecule** | **n** | **Immunostain-negative** | **Immunostain-positive** | | | ***P*-value** |
| --- | --- | --- | --- | --- | --- | --- |
|  |  |  | **Weak** | **Moderate** | **Strong** |  |
| **SEMA3A** |  |  |  |  |  | **0.861** |
| **LN (+)** | **31** | **15** | **13** | **2** | **1** |  |
| **LN (-)** | **25** | **13** | **8** | **3** | **1** |  |
| **SEMA3B** |  |  |  |  |  | **0.189** |
| **LN (+)** | **32** | **14** | **4** | **6** | **8** |  |
| **LN (-)** | **26** | **5** | **7** | **7** | **7** |  |
| **SEMA3C** |  |  |  |  |  | **0.381** |
| **LN (+)** | **34** | **23** | **5** | **3** | **3** |  |
| **LN (-)** | **26** | **12** | **7** | **3** | **4** |  |
| **SEMA3E** |  |  |  |  |  | **0.794** |
| **LN (+)** | **35** | **2** | **8** | **5** | **20** |  |
| **LN (-)** | **25** | **2** | **5** | **6** | **12** |  |
| **PLXNA1** |  |  |  |  |  | **0.069** |
| **LN (+)** | **33** | **19** | **9** | **3** | **2** |  |
| **LN (-)** | **25** | **19** | **2** | **0** | **4** |  |
| **PLXNA2** |  |  |  |  |  | **1.000** |
| **LN (+)** | **35** | **11** | **15** | **6** | **3** |  |
| **LN (-)** | **24** | **7** | **11** | **4** | **2** |  |
| **PLXNA3** |  |  |  |  |  | **0.76** |
| **LN (+)** | **31** | **9** | **13** | **5** | **4** |  |
| **LN (-)** | **24** | **9** | **10** | **4** | **1** |  |
| **PLXND1** |  |  |  |  |  | **0.941** |
| **LN (+)** | **34** | **3** | **12** | **8** | **11** |  |
| **LN (-)** | **26** | **1** | **9** | **6** | **10** |  |
| **NRP1** |  |  |  |  |  | **0.578** |
| **LN (+)** | **34** | **3** | **12** | **8** | **11** |  |
| **LN (-)** | **26** | **1** | **8** | **4** | **13** |  |
| **NRP2** |  |  |  |  |  | **0.22** |
| **LN (+)** | **35** | **28** | **2** | **4** | **1** |  |
| **LN (-)** | **25** | **22** | **3** | **0** | **0** |  |

LN(+): with lymph node metastasis at diagnosis; LN(-): no lymph node metastasis at diagnosis
