# Supplementary material for: SOX4 Transcriptionally Regulates Multiple SEMA3/Plexin Family Members and Promotes Tumor Growth in Pancreatic Cancer
Source: PLoS One. 2012 Dec 12;7(12):e48637. doi: 10.1371/journal.pone.0048637 (PMC3520963; doi:10.1371/journal.pone.0048637)
Supplement: Table S3 — Putative SOX4 and E2F1 binding sites (position from the transcription starting sites). (DOC) [file pone.0048637.s008.doc]

**Supplementary Table S3: Putative SOX4 and E2F1 binding sites (position from the transcription starting sites)**

| **Gene** | **SOX4 sites** | **E2F1 sites** |
| --- | --- | --- |
| ***SEMA3A*** | **-514 ~ -508** | **-897 ~ -890, -173 ~ -166** |
| ***SEMA3B*** | **-979 ~ -973** | **-1591 ~ -1585, -1585 ~ -1578, -1533 ~ -1527, -1236 ~ -1230, -994 ~ -988, -637 ~ -631, -619 ~ -611, -199 ~ -193, -114 ~ -108, -113 ~ -105, -62 ~ -56, -37 ~ -31, -32 ~ -26, -20 ~ -14** |
| ***SEMA3C*** | **-917 ~ -911**  **-845 ~ -839** | **-502 ~ -496, -480 ~ -474, -467 ~ -461, -326 ~ -320, -55 ~ -49, -9 ~ -3** |
| ***SEMA3E*** | **-2488 ~ -2482**  **-1430 ~ -1424** | **None** |
| ***SEMA3F*** | **-1828 ~ -1822**  **+894 ~ +900** | **-1563 ~ -1557, -879 ~ -873, -863 ~ -857, -837 ~ -831, -796 ~ -793, -759 ~ -753, -737 ~ -731, -634 ~ -628, -604 ~ -598, -591 ~ -585, -579 ~ -573, -538 ~ -532, -527 ~ -521, -487 ~ -481, -476 ~ -470, -465 ~ -459, -437 ~ -431, -423 ~ -417, -391 ~ -385, -371 ~ -365, -347 ~ -341, -337 ~ -331, -311 ~ -305, -264 ~ -258, -239 ~ -233, -176 ~ -170, -165 ~ -159, -122 ~ -116, -100 ~ -94, -25 ~ -19, -8 ~ -1** |
| ***PLXNA2*** | **-1515 ~ -1509**  **-1377 ~ -1371** | **-1188 ~ -1182, -976 ~ -970, -946 ~ -940, -469 ~ -463, -227 ~ 0221, -183 ~ -177, -170 ~ -163, -164 ~ -158, -160 ~ -154, -137 ~ -131, -131 ~ -125, -128 ~ -122, -116 ~ -110, -110 ~ -104, -95 ~ -89, -83 ~ -76, -61 ~ -55** |
| ***PLXNA3*** | **-3199 ~ -3193** | **-2082 ~ -2076, -1989 ~ -1983, -1942 ~ -1936, -1648 ~ -1642, -1601 ~ -1595, -1444 ~ -1438, -1221 ~ -1215, -942 ~ -936, -724 ~ -718, -681 ~ -675, -657 ~ -651, -487 ~ -481, _423 ~ -417, -389 ~ -383, -331 ~ -325, -297 ~ -291, -287 ~ -281, -265 ~ -259, -243 ~ -237, -225 ~ -219, -186 ~ -180, -167 ~ -161, -123 ~ -117, -102 ~ -96, -64 ~ -58, -59 ~ -53, -54 ~ -48, -22 ~ -16** |
| ***PLXND1*** | **-1626 ~ -1620** | **-2100 ~ -2094, -1866, -1860, -1584 ~ -1578, -1539 ~ -1533, -1446 ~ -1440, -1153 ~ -1147, -992 ~ -986, -969 ~ -963, -509 ~ -503, -486 ~ -480, -425 ~ -419, -342 ~ -336, -304 ~ -298, -261 ~ -255, -258 ~ -252, -207 ~ -201** |
| ***NRP1*** | **-728 ~ -722** | **-1799 ~ -1793, -1561 ~ -1555, -711 ~ -705, -470 ~ -464, -421 ~ -415** |
